# Oxygen Levels Do Not Determine Radiation Survival of Breast Cancer Stem Cells

**DOI:** 10.1371/journal.pone.0034545

**Published:** 2012-03-29

**Authors:** Chann Lagadec, Carmen Dekmezian, Lucile Bauché, Frank Pajonk

**Affiliations:** 1 Department of Radiation Oncology, David Geffen School of Medicine at UCLA, University of California Los Angeles, Los Angeles, California, United States of America; 2 Jonsson Comprehensive Cancer Center at UCLA, University of California Los Angeles, Los Angeles, California, United States of America; University of South Alabama, United States of America

## Abstract

For more than a century oxygen has been known to be one of the most powerful radiosensitizers. However, despite decades of preclinical and clinical research aimed at overcoming tumor hypoxia, little clinical progress has been made so far. Ionizing radiation damages DNA through generation of free radicals. In the presence of oxygen these lesions are chemically modified, and thus harder to repair while hypoxia protects cells from radiation (Oxygen enhancement ratio (OER)). Breast cancer stem cells (BSCSs) are protected from radiation by high levels of free radical scavengers even in the presence of oxygen. This led us to hypothesize that BCSCs exhibit an OER of 1. Using four established breast cancer cell lines (MCF-7, T47D, MDA-MB-231, SUM159PT) and primary breast cancer samples, we determined the number of BCSCs using cancer stem cell markers (ALDH1, low proteasome activity), compared radiation clonogenic survival and mammosphere formation under normoxic and hypoxic conditions, and correlated these results to the expression levels of key members of the free radical scavenging systems. The number of BCSCs increased with increased aggressiveness of the cancer. This correlated with increased radioresistance (SF_8Gy_), and decreasing OERs. When cultured as mammospheres, breast cancer cell lines and primary samples were highly radioresistant and not further protected by hypoxia (OER∼1).

We conclude that because BCSCs are protected from radiation through high expression levels of free radical scavengers, hypoxia does not lead to additional radioprotection of BCSCs.

## Introduction

In 1909 Gottwald Schwarz reported that a reduction in blood flow and thus oxygen supply protected the human skin from X-rays [1]. Since then a plethora of studies confirmed Schwarz's observation in different species and tissues. Today, oxygen is recognized to be one of the most powerful radiation sensitizers. The ratio of the radiation doses required for equal cell killing under hypoxic and normoxic conditions is called oxygen enhancement ration (OER) and is for most cells and X-rays in the range of 2–3. A widely accepted mechanism behind the sensitizing effects of oxygen is that DNA lesions produced in the presence of oxygen result in chemically modified DNA strands that cannot easily be repaired.

Cancers are known for their irregular vasculature that fails to provide sufficient oxygen supply to parts of a tumor [2], thus leading to chronic hypoxia in a subpopulation of tumor cells distant from capillaries. In addition, high intra-tumoral pressure and the irregular structure of the tumor blood vessels [2] hinders regular blood flow and primes the tumor vasculature to frequent occlusions by micro-thrombosis and subsequent recanalization, thereby causing changes between acute hypoxia and reoxygenation of those cancer cells that depend on the affected capillaries [3]. A general assumption is that during fractionated radiation treatment, normoxic cells are preferentially killed and portions of the surviving chronically hypoxic parts of the tumor are vascularized, oxygenated and thus, sensitized to subsequent fractions of radiation [4]. In addition, fractionated radiation normalizes the irregular structure of the vascular network inside of tumors, thereby reducing the frequency of micro-thrombosis and acute hypoxia [5]. Even though there is some controversy on which form of hypoxia is more important for treatment outcome, it is in general widely accepted that the hypoxic fraction of cells inside a tumor at the time of irradiation determines its curability.

Recent preclinical [6], [7] and clinical [8], [9] data support that solid cancers including breast cancers are organized hierarchically with a small population of cancer stem cells (CSCs), capable of re-growing the entire tumor while their progeny lack this ability [10]. Furthermore, we and others reported that breast CSCs (BCSCs) are relatively resistant to ionizing radiation [11], [12]. One mechanism behind this resistance is a drastically reduced amount of free radicals formed following irradiation, which leads to reduced numbers of DNA double strand breaks, thus suggesting the presence of high levels of free radical scavengers in BCSCs [11]. Based on our observation that BCSCs efficiently scavenge free radicals generated by radiation we hypothesized that BCSCs would not be protected by hypoxia to the same extent as their non-tumorigenic counterparts.

## Results

### High numbers of BCSCs correlate with increased malignancy

**Figure 1:**
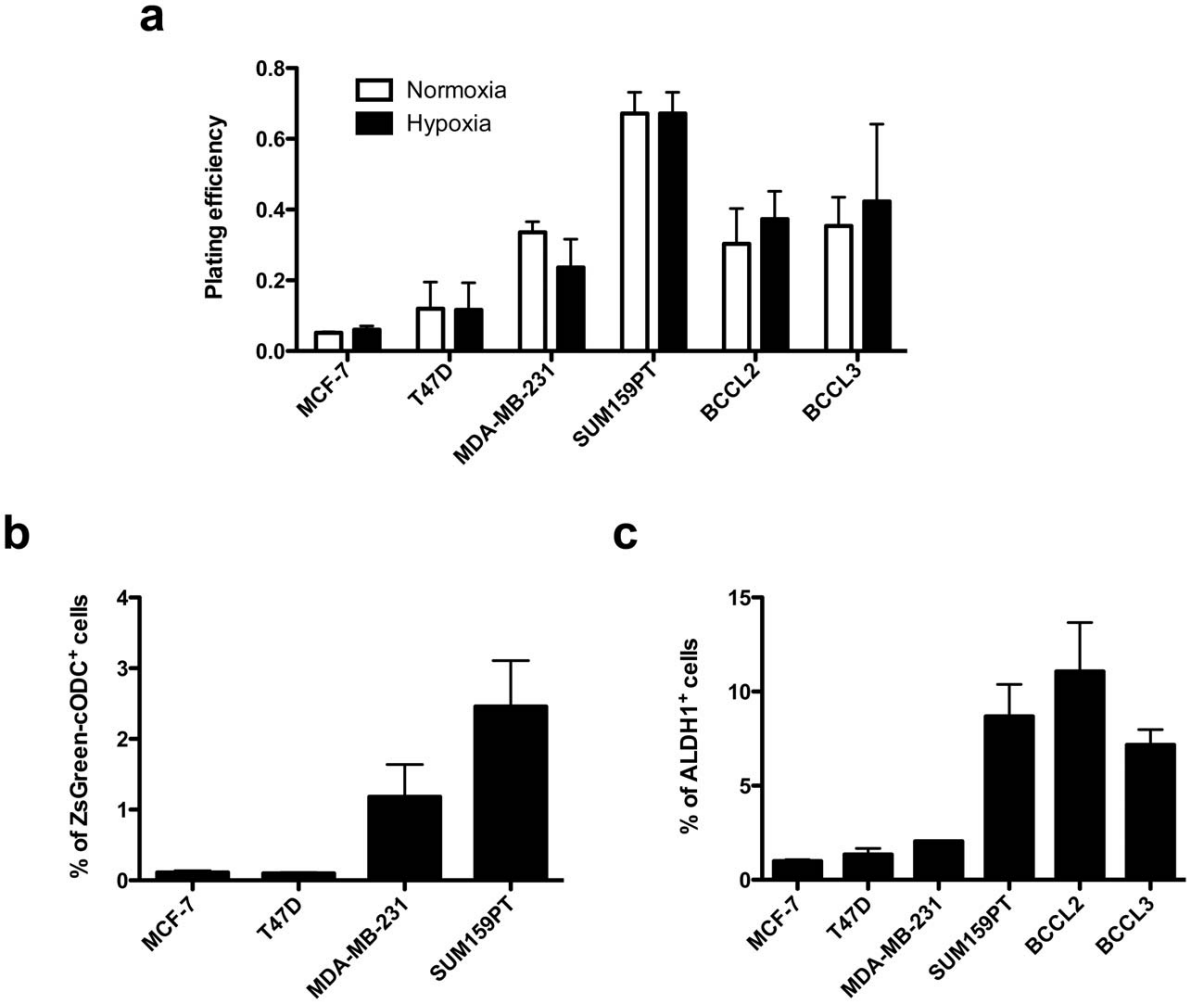
Plating efficiency and percentage of stem cells. (**A**) Plate efficiency of breast cancer cell lines and patient-derived tumor samples in clonogenic survival assays under normoxic and hypoxic conditions. (**B**) Percentage of ZsGreen-cODC^+^ BCSCs in established breast cancer cell lines. (**C**) Percentage of ALDH^+^ cells in established breast cancer cell lines and patient-derived tumor samples.

We started our study comparing plating efficiencies and the size of the putative breast cancer stem cell pool in luminal, basal, and claudin-low breast cancer lines and patient-derived primary breast cancer samples. Plating efficiency (PE) in clonogenic survival assays measures the number of CSCs and transiently amplifying cells. PEs of both luminal lines were low (MCF-7: 5.1±0.5%; T47D: 12±13%) but PEs increased with increased malignancy in basal (MDA-MB-231: 56.5±8.9%), claudin-low cell lines (67.2±13.3%) and primary breast cancer samples from patients with relapsed breast cancers (BCCL2: 38±15.6%; BCCL3: 43±7.1%), thus indicating increased numbers of CSCs and transiently amplifying cells in the more malignant breast cancer types (Fig. 1).

**Figure 2:**
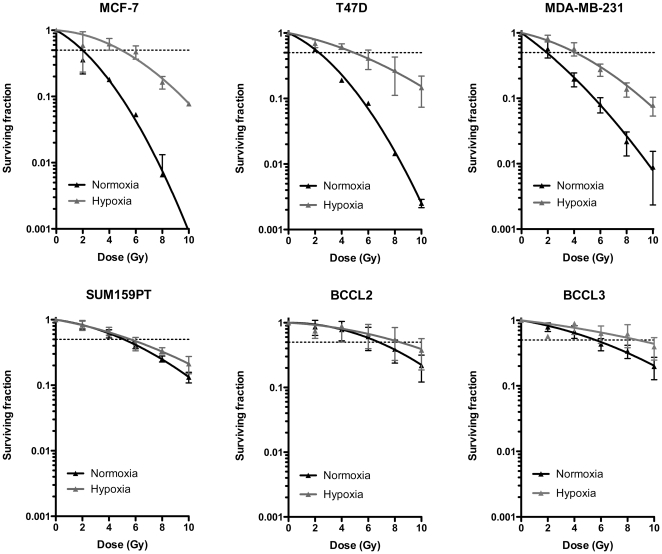
Radiation response of differentiated cells. Clonogenic survival assays of established breast cancer cells lines and primary patient-derived breast cancer samples under normoxic and hypoxic conditions. Data was fitted using a linear quadratic model.

To assess the size of the BCSC pool more directly we used two different marker systems: activity of ALDH1 and a system developed in our laboratory that uses lack of proteasome activity to identify BCSCs [13], [14] and was recently shown to correlate with early recurrence in early stage breast cancer patients (T1/2, N0/1) after radiation therapy [15]. Our system relies on constitutive expression of a fusion protein consisting of the green fluorescent protein ZsGreen and the C-terminal degron of murine ornithine decarboxylase. In cells in which the 26S proteasome is active, the fusion protein is translated and immediately degraded by the 26 proteasome in an ubiquitin-independent fashion. In cells that lack proteasome activity or in which the proteasome is inhibited pharmacologically, the fluorescent fusion protein accumulates and the cells can be identified by fluorescent microscopy or flow cytometry [13]. The frequency of ZsGreen-cODC^+^ BCSCs in the luminal cell lines was 0.1% in MCF-7 and 0.9% T47D. In contrary, the frequency of ZsGreen-cODC^+^ BCSCs in basal MDA-MB-231 cells was 11-fold higher (1.18%) and 25-fold higher in the claudin-low SUM159PT cell line (2.46%) (Fig. 2).

**Figure 3:**
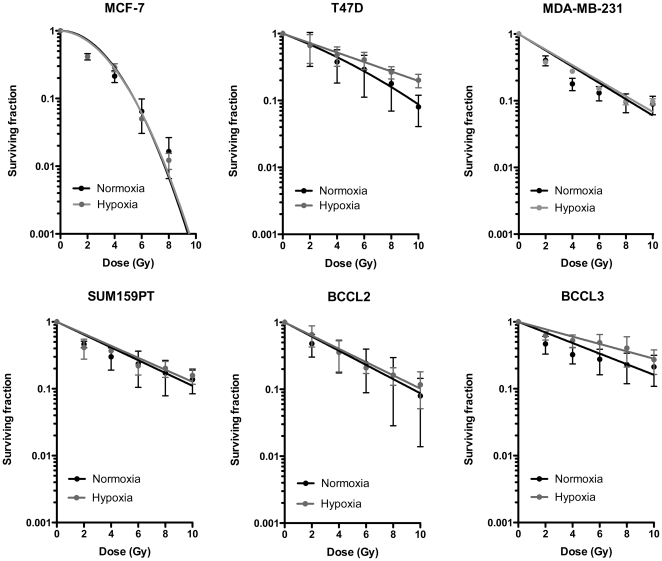
Radiation response of BCSCs. Mammosphere assay using the same cells as in Figure 2. Data was fitted using a linear quadratic model.

Primary patients-derived breast cancer samples do not express the ZsGreen-cODC reporter and engineering these samples to stably express the construct would have required weeks in selections. Since passaging under tissue culture condition carries the risk of induced changes in the phenotype we used ALDH1 activity as an additional marker system to assess the frequency of BCSCs in our cell lines and tumor samples. Results paralleled the trend seen for ZsGreen-cODC^+^ cells with increased frequencies of ALDH^+^ cells in more aggressive breast cancers (MCF-7: 0.98%; T47D: 1.34%; MDA-MB-231: 2.04%; SUM159PT: 8.68%; BCCL2: 11.1%; BCCL3: 7.17%) (Fig. 3).

### Breast cancer cell lines show a broad range of oxygen enhancement ratios

**Figure 4:**
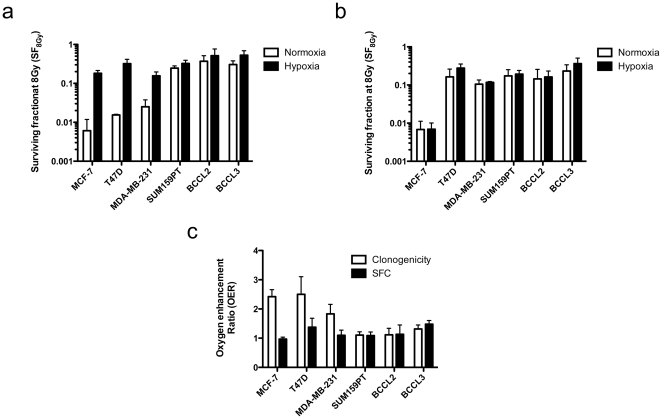
Surviving fractions at 8 Gy and OER. SF_8Gy_ in clonogenic survival assays (**A**) and mammosphere assays (**B**) were calculated. (**C**) Radiation doses required for 50% cell killing were calculated for normoxic and hypoxic conditions and expressed as ratios (hypoxic∶normoxic; oxygen enhancement ratio (OER)).

In order to test our hypothesis we kept cells from four established breast cancer cell lines, MCF-7, T47D, MDA-MB-231, and SUM-159PT under acute hypoxic conditions (2 h, 0.1% O_2_) or normoxia (atmospheric conditions, 21% O_2_) and irradiated cells with 0, 2, 4, 6, 8, or 10 Gy. To assess clonogenic survival, cells were plated and kept under standard tissue culture conditions for 3 weeks after which the number of colonies formed at each dose point was counted and normalized to the corresponding un-irradiated control (Figs. 2, 3, 4C). Since some of the cell lines and patient-derived samples were extremely radioresistant, we calculated OERs at a surviving fraction of 0.5. This allowed for better comparison of the OERs of the different cells. The luminal cell lines MCF-7 and T47D cells showed classical OERs of 2.42±0.34 and 2.5±1.04, respectively while the basal cell line MDA-MB-231 had a slightly lower OER of 1.8±0.73 (Figs. 3 and 4C). This confirmed that we indeed irradiated under hypoxic conditions. However, survival curves for the claudin-low SUM159PT cell line not only revealed the highly radioresistant phenotype of this cell line (Figs. 2, 4A and 4B) but also suggested that radiation survival of clonogenic cells from this cell line did not differ under hypoxic and normoxic conditions (OER = 1.1±0.26) (Figs. 2 and 4C). This suggested a negative correlation of the OER and increasing malignancy of the breast cancer subtype the cell lines were originally derived from.

Next we tested if similar results could be obtained using patient-derived material (BCCL2 and BCCL3). Cells from both samples were highly resistant to radiation (Figs. 2, 4A and 4B) and like in SUM159PT cells, we found only small differences between the survival curves of hypoxic and normoxic cells (BCCL2: OER = 1.1±0.32; BCCL3: OER = 1.3±0.24) (Figs. 2 and 4C).

### Sphere forming breast cancer cells show an OER of 1

Breast cancer cell lines and clinical breast cancer samples contain varying numbers of cells that exhibit a cancer stem cell phenotype [16], [17] and by definition clonogenic survival assays only test for the survival of cells capable of going through at least 5–6 replication cycles, thus measuring the combined survival of cells with short- and long-term proliferative capacity. Sphere-forming assays correlate with self-renewal capacity and tumorigenicity and are as such a better tool to estimate the number of CSCs in a tumor cell population [16]. Mammosphere assays are performed in the absence of fetal calf serum and in the presence of EGF and bFGF [18]. Under these conditions, differentiated cells die from anoikis while BCSCs form mammospheres from single cells if seeded at clonal densities [19]. In order to assess the OER of BCSCs more directly we repeated the above radiation experiment. Instead of performing clonogenic survival assays we seeded the cells into mammosphere media in ultra-low adhesion plate and measured the number of sphere-forming cells after exposure to radiation under normoxic and hypoxic conditions. When cultured as mammospheres, all cells except MCF-7 cells were remarkably radioresistant (Fig. 3). However, the survival curves obtained from experiments performed under hypoxic and normoxic conditions for each individual cell line and for cells from patient samples did not differ and showed an OER close to 1 (Fig. 4C).

### Expression of free radical scavengers and oxygen enhancement ratio in BCSCs

In order to study which part of the free radical detoxification system was upregulated in BCSCs, we performed quantitative RT-PCR for the Foxo family of transcription factors (Foxo1, 3, and 4), key enzymes of the glutathione system (GSS, GCLM, GPX-1), SOD1 and SOD2, the thioredoxin system (TMX1-4) and catalase.

**Figure 5:**
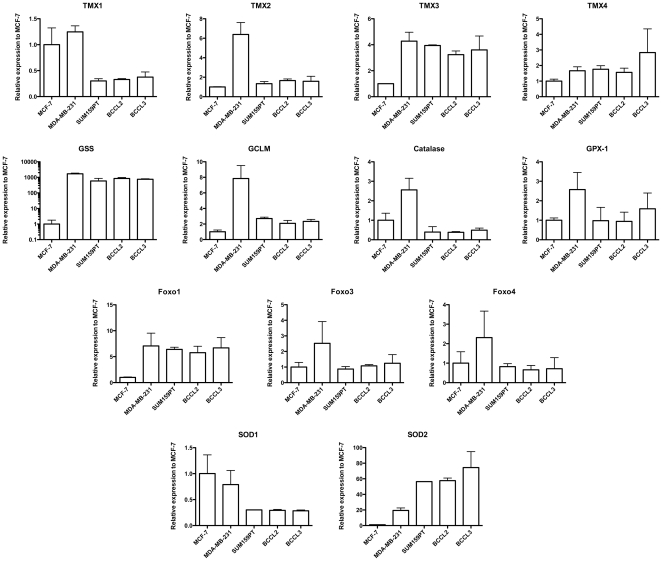
Expression of free radical scavenger mRNAs in breast cancer cells. qRT-PCR using primers for 13 mRNAs involved in free radical scavenging. Expression levels are expressed relative to expression levels found in MCF-7 cells.

In general, more aggressive breast cancers (MDA-MB-231, SUM159PT, BCCL2, BCCL3) had lower levels of TMX1 and SOD1 relative to the luminal MCF-7 cells, and showed up-regulated expression of TMX3, Foxo1, GSS and SOD2 (Fig. 5). However, this picture was not strictly uniform, indicating that different breast cancers may utilize different free radical scavenger systems.

**Figure 6:**
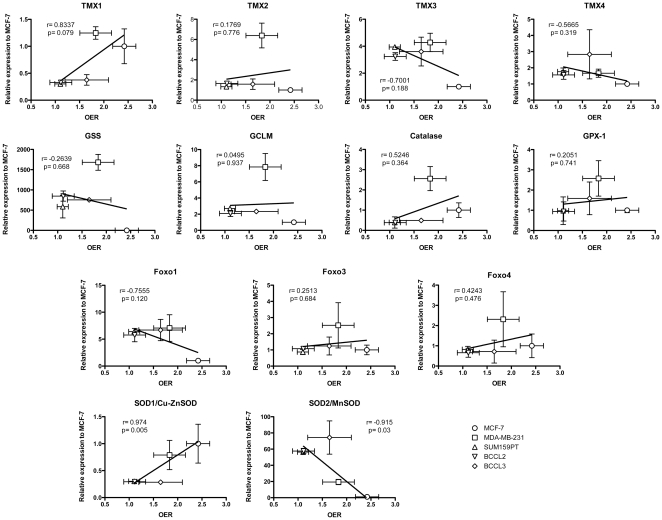
Correlation of free radical scavengers and OER. Correlation of free radical scavenger mRNA expression and oxygen enhancement ratios found in breast cancer cell lines and patient samples.

**Figure 7:**
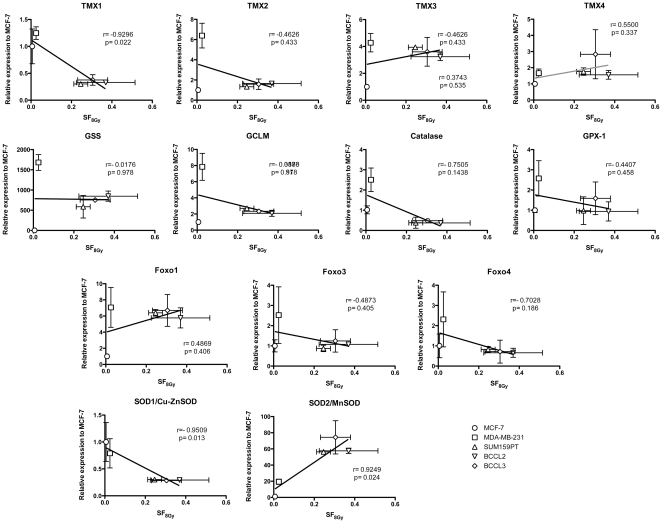
Correlation of free radical scavengers and SF_8Gy_. Correlation of free radical scavenger mRNA expression and SF_8Gy_ found in breast cancer cell lines and patient samples.

When we related the expression levels of the above genes to OER values and the surviving fraction at 8 Gy (SF_8Gy_) we found that low expression levels of SOD1/Cu-ZnSOD (Pearson's correlation, *p* = 0.005) and high expression levels of SOD2/MnSOD (*p* = 0.03) were correlated with a low OER (Fig. 6). A high SF_8Gy_ under normoxic conditions was correlated with low expression levels of SOD1/Cu-ZnSOD (*p* = 0.013), TMX1 (*p* = 0.022) and high expression levels of SOD2/MnSOD (*p* = 0.024) (Fig. 7).

## Discussion

The observation that hypoxia protects cancer cells from radiation [20], [21], [22] and to an even greater extent from many chemotherapeutic drugs [23] has led to decades of experimental and clinical research aimed to improve treatment responses through correction of tumor hypoxia. It is in general accepted that modifying hypoxia improves loco-regional control after radiation treatment. A recent meta-analysis studying 4805 patients with HNSCC treated in 32 randomized clinical trials reported improved loco-regional control (8%), disease-specific (7%) and overall survival (3%) if tumor hypoxia was modified [24]. However, even though this report studied a very heterogeneous group of trials that employed different radiation therapy techniques and strategies to correct tumor hypoxia it is still remarkable that the observed effects were rather moderate. In contrary, differences in loco-regional control between anemic and non-anemic patients with HNSCC, which were attributed to tumor hypoxia, were substantial [25] but correction of tumor anemia through neither blood transfusion [26] nor recombinant erythropoietin [27] corrected the inferior treatment outcome of anemic patients. In the light of an OER of 2–3 for most tumor cells these results suggest that the correction of hypoxia in most of these studies was suboptimal. An alterative interpretation would be that some tumor cells exhibited an intrinsic radioresistance that was independent of hypoxia and that is maintained when cells were re-oxygenated. Our study suggests that in breast cancers such an intrinsically radioresistant tumor cell population indeed exists and that it overlaps with the putative BCSC population. This population is not further protected by hypoxia from radiation doses applied in clinical radiotherapy.

Consistent with our previous observation of reduced radiation-induced free-radical formation in BCSCs [11], cell lines and patients samples that had high numbers of BCSCs had high expression levels of free radical scavenger mRNAs. The overexpression of SOD2/MnSOD, a mitochondria-specific SOD was of particular interest as SOD2/MnSOD is known to protect cells from radiation-induced apoptosis [28] and BCSC do not induce apoptosis in response to radiation [14]. However, the scavenging system used by BCSCs to protect from radiation is most likely not restricted to SOD2/MnSOD and may vary between samples from different patients and even within the different cells of a breast tumor.

Our observations may explain why correcting hypoxia has not improved clinical outcome as dramatically as it affects cell survival experimentally and it raises the question if and how hypoxia contributes to decreased local control after radiation therapy. Furthermore, the reasons for radioresistance are multifactorial, are not restricted to tumor hypoxia alone, and are under modulation by the microenvironment. One possible view is that tumor hypoxia is largely a surrogate marker for more aggressive tumors. However, given the multiple pathways affected by hypoxia this view is rather an oversimplification [29] and even if hypoxia does not affect CSC survival after exposure to ionizing radiation directly, it may still contribute to CSC survival through reprogramming of non-tumorigenic cancer cells into CSCs. Such reprogramming has been described for CD133^−^ non-tumorigenic glioblastoma cells [30] and might be even enhanced during radiotherapy [31] especially if differentiated cells in hypoxic areas are protected from radiation. If this is indeed the case, tumor hypoxia will remain an important factor in clinical oncology and preventing reprogramming of previously non-tumorigenic differentiated cancer cells into CSCs could be an attractive new target in cancer therapy.

## Materials and Methods

### Cell lines

Human SUM159PT breast cancer cell lines were purchased from Asterand (Asterand, Inc., MI). Human MCF-7, T47D, and MDA-MB-231 breast cancer cell lines were purchased from American Type Culture Collection (Manassas, VA). SUM159PT cells were cultured in log-growth phase in F12 Medium (Invitrogen, Carlsbad, CA) supplemented with 5% fetal bovine serum (Sigma Aldrich, St Louis, MO), penicillin (100 units/ml) and streptomycin (100 µg/ml) (both Invitrogen), and insulin (5 µg/mL) and hydrocortisone (1 µg/ml). MCF-7, T47D, and MDA-MB-231 cells were cultured in log-growth phase in Dulbecco's Modified Eagle Medium (DMEM) (Invitrogen, Carlsbad, CA) supplemented with 10% fetal bovine serum, penicillin and streptomycin. All cells were grown in a humidified incubator at 37°C with 5% CO_2_. All cell lines were engineered to express a fusion protein between the green fluorescent protein ZsGreen and the C-terminal degron of murine ornithine decarboxylase as previously described [13].

### Primary Human Breast Cancer Specimens

Anonymized primary tumor specimens were received from the Translational Pathology Core Laboratory (TCPL) and obtained under a protocol approved by the University of California, Los Angeles Institutional Review Boards (IRB#11-002504). BCCL2 is an invasive ductal carcinoma, grade 3, *pT2*, *N0*, *Mx*, ER+, PR−, HER/Neu3+. BCCL3 is an extensive ductal carcinoma in situ (90%), Invasive ductal carcinoma (10%), grade 1, *pT1b*, *N0 (i-)(sn)*, *Mx*, ER−, PR−, HER/Neu−. Both patients were previously treated for breast cancer of the contralateral breast.

### Flow cytometry

BCSCs were identified based on their low proteasome activity [13], [14] using the ZsGreen-cODC reporter system. Cells were trypsinized and ZsGreen-cODC expression was assessed by flow cytometry (Miltenyi Biotech Inc., Auburn, CA) and the FlowJo software package (Tree Star Inc., Ashland, OR. Version 9.3.1). Cells were defined as “ZsGreen-cODC positive” if the fluorescence in the FL-1H channel exceeded the fluorescence level of 99.9% of the empty vector-transfected control cells.

### Aldefluor Assay

The ALDEFLUOR kit (StemCell Technologies, Durham, NC, USA) was used to assess the size of the population with ALDH1 enzymatic activity. Cells were suspended in ALDEFLUOR assay buffer containing ALDH1 substrate (BAAA, 1 µmol/l per 1×10^6^ cells) and incubated for 40 minutes at 37°C. For negative controls an aliquot of each sample of cells was treated with 50 mmol/L diethylaminobenzaldehyde (DEAB), a specific ALDH1 inhibitor. The gates used to identify ALDH1^+^ cells were established using ALDEFLUOR-stained cells treated with DEAB as negative controls. Samples were analyzed using MACSQuant Analyzer flow cytometer and the FlowJo software package.

### Irradiation

Cells grown as monolayers were irradiated at room temperature while inside a hypoxic chamber (Coy Laboratory Products, Grass Lake, MI) using a ^60^Co irradiator (Theratronics T-1000) in 95% N_2_/5% CO_2_ or 95% air/5% CO_2_ at a dose rate of 0.49 Gy/min for the time required to apply a prescribed dose. Corresponding controls were sham irradiated.

### Clonogenic Survival Assays

For clonogenic survival assays, cells were irradiated, trypsinized, counted, and plated into 10 cm Petri dishes using standard culture media. Three weeks later, cells were fixed with 70% ethanol, stained with crystal violet and colonies containing more that 50 cells were counted and normalized to their corresponding un-irradiated control. Data points were fitted using a linear-quadratic model (GraphPad Prism, version 5.0).

### Mammosphere Assay

After irradiation, cells were trypsinized, counted, and plated into mammosphere media (DMEM-F12, 0.4% BSA (Sigma), 10 ml/500 ml B27 (Invitrogen) 5 µg/ml bovine insulin (Sigma), 4 µg/ml heparin (Sigma), 20 ng/ml fibroblast growth factor 2 (bFGF, Sigma) and 20 ng/ml epidermal growth factor (EGF, Sigma)) into 96-well ultra-low adhesion plates. For 0 Gy controls, 256 cells/well were seeded and diluted 1∶1 over the entire plate. For plates subjected to irradiation, the number of cells seeded was adjusted based on their radiation sensitivity. Growth factors, EGF and bFGF, were added every 3 days, and the cells were allowed to form spheres for 20 days. The number of spheres formed per well was then counted manually with a cut-off diameter of 100 µm and expressed as a percentage of the initial number of cells plated. To avoid counting spheres that resulted rather from clumping than from a single clonal cell, only sphere counts within the linear part of the dilution curves were taken into consideration. Three independent experiments were performed.

### Quantitative Reverse Transcription-PCR

Total RNA was isolated using TRIZOL Reagent (Invitrogen). cDNA synthesis was carried out using the SuperScript Reverse Transcription III (Invitrogen). Quantitative PCR was performed in the My iQ thermal cycler (Bio-Rad, Hercules, CA) using the 2× iQ SYBR Green Supermix (Bio-Rad). *C*_t_ for each gene was determined after normalization to GAPDH or RPLP0 and ΔΔ*C*_t_ was calculated relative to the designated reference sample. Gene expression values were then set equal to 2^−ΔΔCt^ as described by the manufacturer of the kit (Applied Biosystems). All PCR primers were synthesized by Invitrogen.

### Statistics

Unless indicated otherwise, at least three biological independent experiments were performed. A *p*-value equal to or less than 0.05 was considered to indicate a statistical significant difference. All data is represented as mean ± standard deviation.
